# Icodextrin *versus* Glucose 2.5% on markers of hypervolemia and survival of patients undergoing automated peritoneal dialysis with an unplanned start: a randomized controlled trial

**DOI:** 10.31744/einstein_journal/2024AO0980

**Published:** 2024-11-12

**Authors:** Leonardo Sotello Azevedo, Vanessa Burgugi Banin, Dayana Bitencourt Dias, Marcela Lara Mendes, Camila Albuquerque Alves, Maryanne Zilli Canedo Silva, Thyago Proença de Moraes, Daniela Ponce

**Affiliations:** 1 Universidade Estadual Paulista Faculdade de Medicina de Botucatu Internal Medicine Department Botucatu SP Brazil Internal Medicine Department, Faculdade de Medicina de Botucatu, Universidade Estadual Paulista, Botucatu, SP, Brazil.; 2 Pontifícia Universidade Católica do Paraná Internal Medicine Department Curitiba PR Brazil Internal Medicine Department, Pontifícia Universidade Católica do Paraná, Curitiba, PR, Brazil.

**Keywords:** Renal insufficiency, chronic, Peritoneal dialysis, Icodextrin, Glucose, Urgent start, Hypervolemia, Survival

## Abstract

**Objective:**

The efficacy of icodextrin *versus* glucose patients undergoing peritoneal dialysis remains unclear. The study was designed to compare the effects of once-daily long-dwell icodextrin *versus* glucose on markers of hypervolemia and survival among patients with kidney failure undergoing an unplanned initiation of automated peritoneal dialysis.

**Methods:**

This was a randomized, non-blinded, and prospective controlled study. Prevalent and stable patients undergoing automated peritoneal dialysis with a recent peritoneal equilibration test showing a dialysate/plasma creatinine of >0.50 were randomized to receive either 7.5% icodextrin or 2.5% glucose solution. Patients were evaluated at baseline (one month after the start of peritoneal dialysis), 3 months, and 6 months after inclusion. The peritoneal dialysis solution was used for at least 3 months, with a follow-up period of 24 months.

**Results:**

Thirty patients were enrolled. There were no baseline differences between the groups. During the study period, patients in the Icodextrin Group showed improvements in the phase angle and ultrafiltration, whereas there were no changes in the Glucose Group. Additionally, extracellular water was significantly lower in the Icodextrin Group at the end of the study than at baseline. No statistical differences between the two groups were observed in urine volume, ultrafiltration, extracellular water, phase angle, renal creatinine clearance, use of diuretics and antihypertensives, or blood pressure. During the 24-month follow-up, the number of events related to overall mortality was seven (Icodextrin Group, n=4; Glucose Group, n=3), with no difference between the groups for this outcome or technique survival.

**Conclusion:**

Icodextrin significantly improved ultrafiltration, extracellular water, and phase angle at the end of the study compared to baseline in patients on the urgent start of automated peritoneal dialysis.

**Registry of Clinical Trials:**

(www.ctri.nic.in) under the number RBR-97z4wh6.

## In Brief

The osmotic characteristics of icodextrin are related to higher ultrafiltration than glucose-based peritoneal dialysis solutions. Consequently, it is associated with an improvement in the biomarkers of volume overload in patients undergoing automated peritoneal dialysis at an unplanned start. However, the mortality and technique survival rates did not differ with glucose use.

## Highlights

■ No baseline differences between the groups icodextrin versus glucose.
■ Icodextrin significantly improved ultrafiltration, extracellular water, and phase angle.
■ Mortality and technique survival did not differ between the groups during follow-up.

## Graphical abstract

**Figure:**
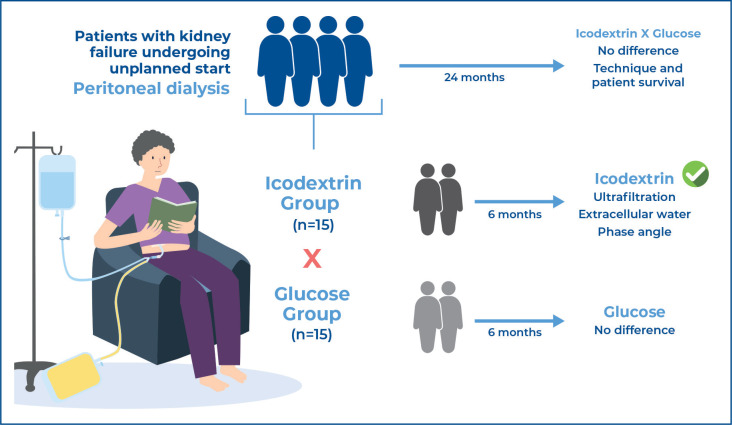

## INTRODUCTION

Icodextrin, a high molecular weight glucose polymer, has become an essential osmotic agent in peritoneal dialysis (PD) solutions over the past few decades. It plays a crucial role in PD treatment by creating an osmotic gradient that removes excess fluid and waste products from the blood. These properties have been associated with better control of volume overload, which is a common finding in patients undergoing dialysis. Given its osmotic characteristics, higher ultrafiltration is obtained compared with glucose-based PD solutions; consequently, an improvement in biomarkers associated with volume overload is anticipated.^(1–5)^

Despite its increasing prevalence in clinical practice, there is a lack of randomized clinical trials investigating the efficacy and safety of icodextrin compared to other fields of medicine. In particular, the main interest of most RCTs is ultrafiltration, and only a few studies provide information on other important markers of hypervolemia, such as blood pressure and bioimpedance data, including phase angle (PA) and extracellular water (ECW).

## OBJECTIVE

To compare the effects of once-daily long-dwell icodextrin versus glucose on markers of hypervolemia and survival among patients with kidney failure undergoing unplanned automated peritoneal dialysis.

## METHODS

### Patients

This prospective randomized controlled clinical trial was conducted at the *Hospital das Clínicas of Faculdade de Medicina de Botucatu*, São Paulo, Brazil. Adult patients were eligible and included in the study if they were prevalent patients with stage 5 chronic kidney disease (CKD) on automated PD, started urgent PD, and had a recent peritoneal equilibration test (PET) showing a dialysate/plasma creatinine level >0.50. The exclusion criteria were age <18 or >80 years, urine volume <400ml/d, urinary tract obstruction due to neoplasm, neurogenic bladder, pregnancy, and previous renal replacement therapies, including PD, HD, and kidney transplantation.

### Study protocol

A total of 30 patients with prevalent PDs from the *Faculdade de Medicina de Botucatu* were enrolled from September 2021 to March 2022. They were randomly assigned to a Glusose Group (n=15) treated with a maximum of 10L of 1.5% or 2.5% daily (Dianeal, Baxter) or an Icodextrin Group (n=15) treated with a maximum of 8L of 1.5% or 2.5% Dianeal in association with a daytime dwell of 2 or 1.5L of 7.5% icodextrin-containing solution (Extraneal, Baxter) for at least 3 months. The patients were evaluated at baseline (one month after the start of PD), 3 months, and 6 months after inclusion, and the follow-up period was 24 months.

The study was conducted in accordance with the ethical principles of the Declaration of Helsinki and was approved by the *Faculdade de Medicina* *de Botucatu* Research Ethics Committee (CAAE: 28325020.5.0000.5411; #3.936.169). All the patients provided written informed consent to participate in the study.

### Clinical efficacy and outcomes

The primary outcome was the improvement in markers of hypervolemia such as ultrafiltration, ECW, PA, and blood pressure of patients undergoing CCPD with an unplanned start after 3 and 6 months. The secondary objectives were the rates of technical and patient survival at 6 months after PD. Technical survival was defined as the discontinuation of PD therapy owing to volume overload and infectious or mechanical complications. Clinical indices, including markers of body fluid status and RRF, were measured using daily urine volume, renal creatinine clearance, and weekly Kt/V, which were measured at one (baseline), three, and six months after the initiation of urgent PD.

A PET was performed at 1 and 6 months to measure the peritoneal transport status^(3)^ with a 4-hour dwell period using a 2.5% glucose concentration and 2L volume exchange. The D/P creatinine ratio was measured after the 4-hour dwell period.

Weekly Kt/V urea was calculated from a 24-hour collection of dialysate and urine samples. RRF was calculated as the average residual renal creatinine and urea clearance. Markers of fluid status were evaluated using single-frequency bioimpedance analysis (Biodynamics model 450), which measures resistance, reactance, and PA and estimates total body water and intra-and extracellular water.

### Statistical analyses

Continuous variables were expressed as either mean and standard deviation or median and interquartile range, depending on the distribution. Categorical variables were expressed as frequencies and percentages. Comparison of continuous variable values between the Icodextrin and Glucose Groups was performed using Student's *t*-test and Mann-Whitney *U* test. Comparison of ordinal variables, such as PET, use of diuretics, and antihypertensives was evaluated using the χ^2^ test.

We used repeated-measures ANOVA to assess changes within the group for clinical variables and examined the sphericity assumption using Mauchly's test (F ratio). If the sphericity assumption was violated, which was the case only for diastolic blood pressure, we used the recommended Greenhouse-Geisser epsilon value to test for significance. To compare the impact of the intervention on different clinical outcomes, we calculated the Z-score (individual value- mean/standard deviation) for each outcome and assessed the delta between Time 6 and Time 0 using ANOVA.

For survival analysis, we used the log-rank test to compare outcomes and plotted a Kaplan- Meier curve for overall mortality, technique failure, and dropout of any cause. We defined censoring for overall mortality and technique failure as any event that was not the primary outcome of interest as well as patients who remained active at the end of the study. Censoring for the outcome of dropout was restricted to patients who remained active at the end of the study. Finally, owing to the sample size of our study, and to facilitate the visualization of individual variations, we created scatter plots with the T0 and T6 values for each subgroup.

## RESULTS

**Figure 1:**
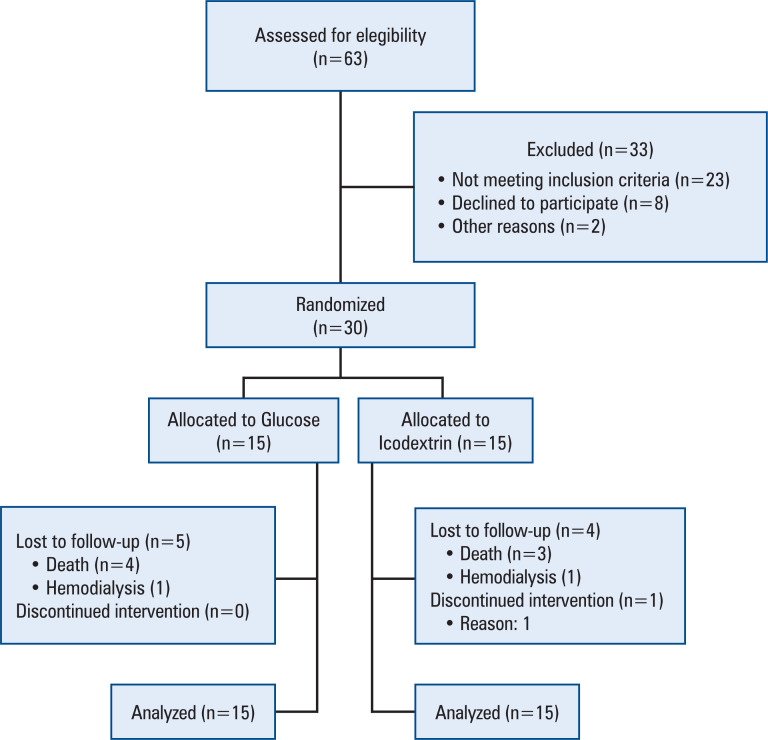
Flow-chart of patient inclusion

Thirty patients were enrolled in the study; 15 were randomized to the Icodextrin Group and 15 to the Glucose Group (Figure 1). At baseline, the Icodextrin and Glucose Groups were similar in all analyzed variables, including clinical characteristics, etiology of CKD, time on automated peritoneal dialysis (APD), characteristics of peritoneal transport, laboratory measurements, blood pressure, dialysis efficacy, use of diuretics and antihypertensives, PA, and ECW (Table 1).

**Table 1:** Clinical and demographic characteristics of the study population at the baseline

Variable			Glucose Group n=15	Icodextrin Group n=15	Overall	p value
Demographic						
Age (years)			56.5±16.1	56.1±15.3	56.3±15.4	0.94
Sex (male)			7 (46.7)	3 (20)	10 (33.3)	
Etiology of chronic Kidney disease [n (%)]						0.58
	Diabetes		6 (40)	3 (20)	9 (30)	
	Hypertension		2 (13.3)	5 (33.3)	7 (23.3)	
	Glomerulonephritis		1 (6.7)	2 (13.3)	3 (10)	
	Others		2 (13.3)	2 (13.3)	4 (12.3)	
	Unknown		4 (26.7)	3 (20)	7 (23.3)	
Clinical						
	Dialisate/Plasma creatinine [n (%)]					0.18
		High	3 (23.1)	7 (58.3)	10 (40)	
		High average	8 (61.5)	3 (25)	11 (44)	
		Low average	2 (15.4)	2 (16.7)	4 (16)	
	Kt/V (total)		1.80±0.53	2.28±0.82	2.0±0.7	0.07
	Kt/V (renal)		0.43(0-0.84)	0.75(0.14-1.01)	0.54 (0-0.98)	0.16
	Systolic blood pressure (mmHg)		138.5±22.8	148.4±25.5	143.5±24.3	0.27
	Diastolic blood pressure (mmHg)		77.1±16.3	85.3±16.8	81.2±16.8	0.19
	Extracellular water (L)		20.5±5.0	23.4±11.5	22.0±8.8	0.36
	Ultrafiltration (L)		1134±611	1083±530	1112±565	0.82
	Residual kidney function (ml)		805 (0–1500)	352(0–500)	762 (0–1000)	0.10
Use of medications [n(%)]						0.08
	Loop diuretics (furosemide)*		15 (100)	15 (100)	30 (100)	
	Diuretics		3 (20)	3 (20)	6 (20)	
		ACEI + Diuretics	7 (46)	6 (40)	13 (43.3)	
		ACEI + Diuretics + CCB	5 (33)	4 (36)	9 (30)	
		ACEI + Diuretics + BB	-	2 (13.3)	2 (6.7)	
	Phase angle (°)		5.08 ± 1.36	5.04 ±1.12	5.51±1.34	0.83

* Dose of 160 to 320mg/day.

ACEI: angiotensin-converting enzyme inhibitor; CCB: calcium channel blocker; BB: beta-blocker.

**Figure 2:**
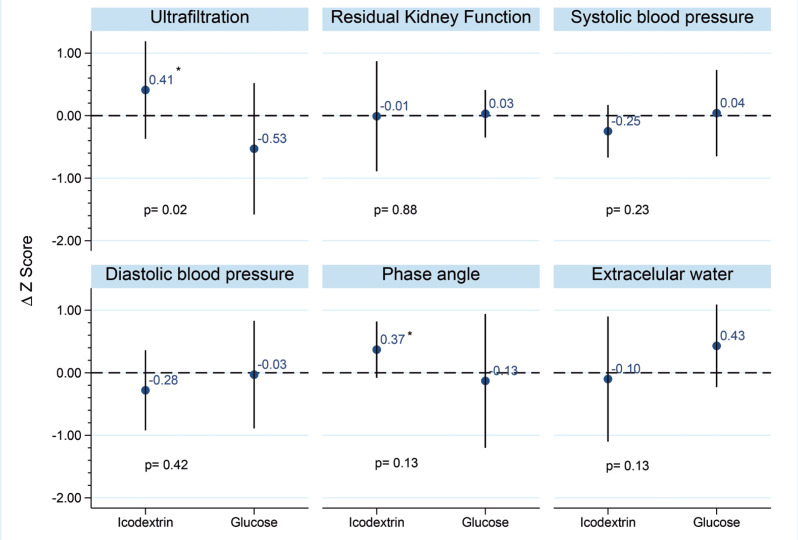
Comparison of changes in Z-scores of different clinical outcomes from baseline to 6 months between groups

**Figure 3:**
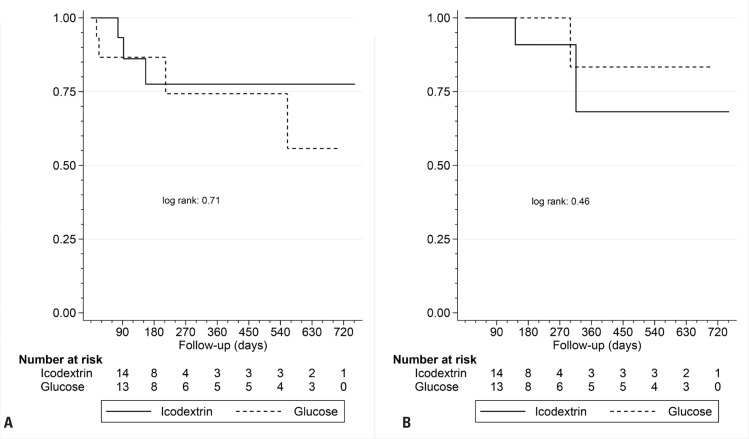
Patient (3A) and Technique Survival (3B) - Kaplan-Meier

During the study period, patients in the Icodextrin Group showed improvements in the PA and UF, whereas there were no changes in the Glucose Group (Tables 2 and 3). Additionally, ECW was significantly lower in the Icodextrin Group at the end of the study than at baseline (Tables 2 and 3). No statistical differences between the two groups were observed in urine volume, UF, ECW, PA, renal creatinine clearance, or BP (Figure 2). During the follow-up period of 24 months, the number of events related to overall mortality was seven (Icodextrin Group, n=4; Glucose Group, n=3), and two events were reported as failure of technique (Icodextrin Group n=1, Glucose Group n=1) (Tables 1S and 2S, Supplementary Material). No differences were observed in the technique or patient survival (Figures 3A and 3B).

**Table 2:** Clinical outcomes at baseline, T3 and T6

	Baseline		T3 (after 3 months)		T6 (after 6 months)	
	Icodextrin Group	Glucose Group	Icodextrin Group	Glucose Group	Icodextrin Group	Glucose Group
Phase angle (°)	5.04±1.12*	5.08±1.36	4.5 (4.1-6.4)	5.7 (5.4-7.0)	5.8±1.0*	5.6±1.3
Extracellular water (L)	23.4±11.5*	20.5±5.0	19.1±3.9	19.4±3.6	16.4±7.0*	20.2±5.3
Diastolic blood pressure (mmHg)	85.3±16.8	77.1±16.3	87.3±18.0	85.3±18.6	82.6±15.0	81.2±18.4
Systolic blood pressure (mmHg)	148.4±25.5	138.5±22.8	137.5±18.8	146.6±29.3	141.1±19.3	138.4±23.2
Residual diuresis	352 (0–500)	805 (0–1500)	325 (0–1500)	50 (0–350)	0 (0–830)	0 (200–1200)
Kidney Kt/V	0.75 (0.14–1.01)	0.43 (0–0.84)	0.63 (0–0.98)	0.66 (0.42–0.94)	0.21 (0–0.98)	0.66 (0.42–0.94)
Total Kt/V	2.28±0.82	1.80±0.53	2.3±0.92	1.9±0.50	2.35±0.94	1.97±0.50
Ultrafiltration (L)	1083±530*	1134±611	1200 (515–1300)	615 (195–1218)	1296 (1050–1621)	807 (345–1058)

* p<0.05.

**Table 3:** Changes within groups in different clinical parameters along the study

	F ratio		Greenhouse-Geisser épsilon (p value)*	
	Icodextrin Group	Glucose Group	Icodextrin Group	Glucose Group
Phase angle (°)	0.82	0.68	0.04	0.39
Extracellular water (L)	2.37	0.57	0.05	0.56
Diastolic blood pressure (mmHg)	0.87	1.80	0.41	0.19
Systolic blood pressure (mmHg)	3.31	0.84	0.07	0.40
Residual diuresis	2.95	1.80	0.08	0.20
Kidney KtV	0.73	0.76	0.42	0.48
Total KtV	0.77	0.17	0.41	0.71
Ultrafiltration (L)	5.41	2.58	0.02	0.12

* Repeated-measures analysis of variance (ANOVA).

## DISCUSSION

In this RCT, we showed that the Icodextrin Group presented with increased UF, higher PA, and reduced ECW at the end of the study compared with baseline, whereas these parameters were unaltered in the Glucose Group. There were no differences in the Icodextrin Group and Glucose Group in terms of urine volume, UF, ECW, PA, renal creatinine clearance, use of diuretics and antihypertensives, BP, technique, or patient survival.

A previous study with a large cohort of incident patients on PD in different countries showed that the majority of patients started PD with volume overload (33% moderate and 24% severe), and the use of hypertonic PD solutions (defined as at least one exchange with a dextrose concentration >1.5%) at baseline was 31%, which increased to 51% after three years.^(6)^ In this context, the use of icodextrin should be discussed. Our findings are consistent with those of previous studies and with a recent systematic review showing that icodextrin resulted in increased UF and fewer episodes of fluid overload.^(4,5,7–15)^

There was no benefit of icodextrin in terms of the PD technique and patient survival. However, these outcomes are confounded by the small number of patients included and by interventions or contamination between groups. Our findings are in contrast with those of observational studies, which generally showed improved technique and patient survival with icodextrin treatment.^(16–19)^

Icodextrin reduces the daily exposure of the peritoneum to glucose compared to conventional glucose solutions, with a greater peritoneal biocompatibility. Despite this, we observed no difference in peritoneal solute transport, peritoneal small-solute clearance, and technique survival between the ICO- and GLU-only PD regimens. A single exchange of icodextrin per day instead of glucose may be clinically insufficient as a PD regimen with enhanced biocompatibility.^(20,21)^

This study had a few limitations. Firstly, it was performed at a single center and included a small number of patients. Secondly, carbohydrate metabolism was not evaluated in this study. Despite these limitations, this was the first study to evaluate the role of the ICO on markers of hypervolemia and survival of patients undergoing APD with an unplanned start.

Therefore, there is an urgent need to improve PD outcomes and reduce associated costs. Icodextrin provides an opportunity to decrease important complications such as fluid overload. Based on our updated results, increased access to icodextrin is likely to benefit patients worldwide, and multicenter RCT should be implemented.

## CONCLUSION

In conclusion, our study demonstrated the clinical benefits of icodextrin due to improved ultrafiltration, extracellular water, and phase angle at the end of the study compared with the baseline in patients on urgent initiation of automated peritoneal dialysis.

## Data Availability

The datasets used and/or analyzed in the current study are available from the corresponding author upon reasonable request.

## SUPPLEMENTARY MATERIAL Icodextrin *versus* Glucose 2.5% on markers of hypervolemia and survival of patients undergoing automated peritoneal dialysis with an unplanned start: a randomized controlled trial

Leonardo Sotello Azevedo, Vanessa Burgugi Banin, Dayana Bitencourt Dias, Marcela Lara Mendes, Camila Albuquerque Alves, Maryanne Zilli Canedo Silva, Thyago Proença de Moraes, Daniela Ponce

**Table 1S:** Number of events related to the overall mortality

Interval	Beg	Deaths	Lost	Survival	Standard error	[95%CI]
	Total					
16-17	30	1	0	0.9667	0.0328	0.7861-0.9952
23-24	29	1	0	0.9333	0.0455	0.7589-0.9829
77-78	28	1	0	0.9000	0.0548	0.7212-0.9666
90-91	27	0	1	0.9000	0.0548	0.7212-0.9666
93-94	26	1	0	0.8654	0.0627	0.6799-0.9473
111-112	25	0	1	0.8654	0.0627	0.6799-0.9473
112-113	24	0	2	0.8654	0.0627	0.6799-0.9473
132-133	22	0	1	0.8654	0.0627	0.6799-0.9473
143-144	21	0	1	0.8654	0.0627	0.6799-0.9473
156-157	20	1	0	0.8221	0.0729	0.6213-0.9226
168-169	19	0	1	0.8221	0.0729	0.6213-0.9226
171-172	18	0	2	0.8221	0.0729	0.6213-0.9226
196-197	16	0	1	0.8221	0.0729	0.6213-0.9226
202-203	15	0	1	0.8221	0.0729	0.6213-0.9226
213-214	14	1	0	0.7634	0.0883	0.5353-0.8899
242-243	13	0	3	0.7634	0.0883	0.5353-0.8899
300-301	10	0	1	0.7634	0.0883	0.5353-0.8899
316-317	9	0	1	0.7634	0.0883	0.5353-0.8899
529-530	8	0	1	0.7634	0.0883	0.5353-0.8899
560-561	7	1	0	0.6543	0.1262	0.3556-0.8403
569-570	6	0	1	0.6543	0.1262	0.3556-0.8403
689-690	5	0	1	0.6543	0.1262	0.3556-0.8403
690-691	4	0	1	0.6543	0.1262	0.3556-0.8403
703-704	3	0	1	0.6543	0.1262	0.3556-0.8403
717-718	2	0	1	0.6543	0.1262	0.3556-0.8403
752-753	1	0	1	0.6543	0.1262	0.3556-0.8403

**Table 2S:** Number of events related to the technique survival

Interval	Beg	Deaths	Lost	Survival	Standard error	[95%CI]	
	Total						
16-17	30	0	1	10.000	0.0000	.	.
23-24	29	0	1	10.000	0.0000	.	.
77-78	28	0	1	10.000	0.0000	.	.
90-91	27	0	1	10.000	0.0000	.	.
93-94	26	0	1	10.000	0.0000	.	.
111-112	25	0	1	10.000	0.0000	.	.
112-113	24	0	2	10.000	0.0000	.	.
132-133	22	0	1	10.000	0.0000	.	.
143-144	21	1	0	0.9524	0.0465	0.7072-0.9932	
156-157	20	0	1	0.9524	0.0465	0.7072-0.9932	
168-169	19	0	1	0.9524	0.0465	0.7072-0.9932	
171-172	18	0	2	0.9524	0.0465	0.7072-0.9932	
196-197	16	0	1	0.9524	0.0465	0.7072-0.9932	
202-203	15	0	1	0.9524	0.0465	0.7072-0.9932	
213-214	14	0	1	0.9524	0.0465	0.7072-0.9932	
242-243	13	0	3	0.9524	0.0465	0.7072-0.9932	
300-301	10	1	0	0.8571	0.0996	0.5091-0.9654	
316-317	9	1	0	0.7619	0.1261	0.4081-0.9208	
529-530	8	0	1	0.7619	0.1261	0.4081-0.9208	
560-561	7	0	1	0.7619	0.1261	0.4081-0.9208	
569-570	6	0	1	0.7619	0.1261	0.4081-0.9208	
689-690	5	0	1	0.7619	0.1261	0.4081-0.9208	
690-691	4	0	1	0.7619	0.1261	0.4081-0.9208	
703-704	3	0	1	0.7619	0.1261	0.4081-0.9208	
717-718	2	0	1	0.7619	0.1261	0.4081-0.9208	
752-753	1	0	1	0.7619	0.1261	0.4081-0.9208	
